# Capsid Structure of the Fish Pathogen Syngnathus Scovelli Chapparvovirus Offers a New Perspective on Parvovirus Structural Biology

**DOI:** 10.3390/v17050679

**Published:** 2025-05-06

**Authors:** Judit J. Penzes, Jason T. Kaelber

**Affiliations:** 1 Institute for Quantitative Biomedicine, Rutgers, The State University of New Jersey, Piscataway, NJ 08812, USA; jason.kaelber@rutgers.edu; 2 Department of Entomology, Texas A&M University, College Station, TX 77845, USA

**Keywords:** cryo-EM, parvovirus, virus structure, virus evolution, fish pathogen, capsid structure, ssDNA virus, chapparvovirus

## Abstract

Chapparvoviruses (ChPVs) comprise a divergent lineage of the *Parvoviridae* ssDNA virus family and evolved to infect vertebrate animals independently from the *Parvovirinae* subfamily. Despite being pathogenic and widespread in environmental samples and metagenomic assemblies, their structural characterization has proven challenging. Here, we report the first structural analysis of a ChPV, represented by the fish pathogen, Syngnathus scovelli chapparvovirus (SsChPV). We show through the SsChPV structure that the lineage harbors a surface morphology, subunit structure, and multimer interactions that are unique among parvoviruses. The SsChPV capsid evolved a threefold-related depression of α-helices that is analogous to the β-annulus pore of denso- and hamaparvoviruses and may play a role in monomer oligomerization during assembly. As interacting β-strands are absent from the twofold symmetry axis, the viral particle lacks the typical stability and resilience of parvovirus capsids. Although all parvoviruses thus far rely on the threading of large, flexible N-terminal domains to the capsid surface for their intracellular trafficking, our results show that ChPVs completely lack any such N-terminal sequences. This led to the subsequent degradation of their fivefold channel, the site of N-terminus externalization. These findings suggest that ChPVs harbor an infectious pathway that significantly deviates from the rest of the *Parvoviridae*.

## 1. Introduction

Parvoviruses (PVs) encompass the vast and diverse single-stranded DNA (ssDNA) virus family, the *Parvoviridae*, with 188 recognized species in three subfamilies, infecting animals of all major eumetazoan lineages [1]. The family is characterized by a linear ssDNA genome of 3.4 to 6.3 kb in length, packaged into non-enveloped icosahedral virions of 20–28 nm in diameter. The genome, from left to right, is composed of a non-structural (*rep*) and a structural (*cap*) expression cassette, which encode a varied number of non-structural (NS) and structural (VP) proteins, respectively, in a collateral order [1,2]. In addition, genus-specific ancillary proteins may also be present. The coding region of the genome is flanked by hairpin-like, partially double-stranded DNA secondary structures, which are essential for replication and packaging [2,3]. Despite these synapomorphies, only the superfamily 3 helicase (SF3) domain of NS1, which is ~200 amino acid (aa) long, exhibits detectable protein sequence homology throughout the entire family [4].

The *cap* gene expresses 1–4 VPs, which are essentially N-terminal extensions of each other, sharing a common C-terminal segment. The icosahedral shell is assembled by 60 subunits of these C-terminal regions. Typically, the VP possessing the shortest N-terminus is incorporated in the highest copy number into the assembly and considered the major capsid protein [5]. The N-termini of all VPs have been found to be flexible and therefore challenging to characterize structurally. Consequently, these are absent from all PV capsid structures resolved to date. Currently, 177 PV capsid structures have been deposited into the RCSB Protein Data Bank (PDB), 165 of which belong to members of the *Parvovirinae* subfamily (source: https://viperdb.org/, accessed on 1 May 2025). All PV capsids possess *T* = 1 icosahedral symmetry, regardless of the large fluctuations in PV genome size and virion diameter [5]. Each subunit displays an eight-stranded (βB to βI) jelly roll fold as the subunit core [6], in which long loops connect the β-strands to compose the variable capsid’s surface morphology [5]. These are typically referred to by the name of the two β-strands they connect, e.g., the HI loop links βH and βI. With the exemption of Peneaus monodon metallodensovirus (PmMDV), the interior-facing BIDG sheet is complemented by an additional N-terminal β-strand, designated as βA [5,7]. Similar to the *T* = 1 capsids of certain *Cressdnaviricota* phylum members, PV VPs are capable of spontaneous oligomerization and full capsid assembly even without the aid of the viral genome [8,9]. The fivefold symmetry axis of the PV capsid characteristically includes a pore-like opening that continues in a channel and a portal proposed to aid genome packaging and uncoating, as well as serving as the location through which the flexible N-terminal domains can be externalized to the capsid surface [7,10,11,12,13,14].

Two independently evolved parvoviral lineages have been identified to infect vertebrates [4]. One of these comprises the most well-characterized lineage of the family as a distinct subfamily of clear monophyletic origin, the *Parvovirinae*. Members are known to be significant pathogens of humans and other primates, livestock, wildlife, and pets [15]. The subfamily also includes adeno-associated viruses (AAVs), which are prominent human gene therapy vectors [16].

The other lineage is collectively termed chapparvoviruses (ChPVs) after the acronym of the host taxa, which they were initially derived from; **Ch**iroptera, **A**ves, and **P**orcine, respectively. Although near as numerous as the *Parvovirinae*, the existence of chapparvoviruses remained unnoticed until the widespread accessibility of next-generation sequencing methods. The first representatives were derived only in the mid-2010s [17,18,19,20], as opposed to the early 1960s discovery of the exclusively invertebrate-infecting subfamily, the *Densovirinae* [21], and the aforementioned *Parvovirinae* [22]. ChPVs are among the most frequently detected PVs of metagenomic assemblies derived from feces and various bodily fluids, demonstrating an immense host spectrum of all major vertebrate lineages [23,24,25,26,27,28]. ChPVs have been shown to be pathogenic in rodents and primates, exhibiting a nephrotropic pathology [25,29]. Mouse kidney parvovirus (MKPV) is the most well-studied member of the lineage; its transcription strategy and its detailed pathology have been characterized [29].

Currently, ChPVs are classified within the third, extremely heterogenous, subfamily of the *Parvoviridae*, the *Hamaparvovirinae*, within which they encompass two genera, *Chaphamaparvovirus* and *Ichthamaparvovirus*, infecting mammals, birds, reptiles, and fish [4]. Besides phylogenetic evidence, the monophyly of these two genera is supported by their common genome organization, homologous VPs and NSs, shared ancillary proteins, and terminal hairpin morphology [30]. All ChPVs package a rather small genome of 4–4.5 kb in length. The discovery of ChPV-like endogenous viral elements (EVEs) in various arthropod genomes of arachnid, chilopod, coleopteran, and dipluran origin showed that the clade also infects arthropods and in fact may be of arthropod origin altogether [30].

Here, we report the first capsid structure of a ChPV, derived from Syngnathus scovelli chapparvovirus (SsChPV). SsChPV is a pathogen of the gulf pipefish (*Syngnathus scovelli*) and is a member of the exclusively fish-infecting ChPV genus, the *Ichthamaparvovirus*. Through the SsChPV capsid structure, we will show that ChPVs exhibit only a single VP, which lacks the canonical disordered N-terminal segment of parvoviral structural proteins. Furthermore, we revealed that the ChPV capsid architecture deviates significantly from that of other PVs with a distinct capsid surface, a lack of a well-defined fivefold channel, and a weak twofold symmetry axis, which contributes to a lack of virion resilience. Additionally, the ChPV threefold symmetry axis is covered by an opening, analogous to the β-annulus structure shown to be ubiquitous in other hamaparvoviruses and in the *Densovirinae*, which may imply convergent assembly mechanisms.

## 2. Materials and Methods

### 2.1. VLP Expression and Culturing Conditions

The complete coding sequence of SsChPV was artificially synthesized by a commercial gene synthesis service based on the deposited nucleotide sequence under GenBank accession number MN049932. The synthesized sequence upon receipt was verified and cloned into a pFastBac dual promoter-knock-out plasmid [7], utilizing one restriction site for each multiple cloning site, *KpnI* and *SpeI*, respectively. This allowed us to rely on the cis-regulatory elements of the coding genome alone. PCR primers were designed to amplify the VP (5′-GCGACTAGTAACAAACCGCGAATAATGAAAT-3′ and 5′-GCGAAGCTTTCCAGCCCCGGGTGTTGTCCTCTA-3′) and the putative structural protein (SP) (5′-GCGACTAGTCGCTAAAGAAGAAATATGGCTG-3′ and 5′-GCGAAGCTTGCCTTTATTGCTTTTACATGAGAT-3′) protein coding ORFs, respectively. Both PCR products were cloned into a pFastBac1 backbone. Bacmids of the Autographa californica multiple nucleopolyhedrosis virus genome were transformed by the generated pFastBac constructs in DH10Bac chemically competent cells (Thermo Fisher, Waltham, MA, USA). Successful transformation was checked by PCR, utilizing the forward M13 primer paired with the respective reverse PCR primers, targeting the translocation site of the bacmid.

Sf9 cells (ATCC CRL-1711) were obtained directly from the American Type Culture Collection. Suspension cultures were maintained in SF900 II medium (Gibco, Waltham, MA, USA) in a serum-free system at 28 °C. Cellfectin II Reagent (Invitrogen, Waltham, MA, USA) was used for DNA transfection at a cell density of 8 × 10^5^ per well. The culturing medium was aspired and replaced by a seeding medium made of Grace’s complete insect medium supplemented with 5% FBS (Gibco) and Grace’s unsupplemented insect medium, mixed at a ratio of 1:6, respectively. After adding the transfection reagent–DNA mixture to the wells, cells were incubated for 5 h at 28 °C. The aspired transfection medium was replaced with SF900 II medium. Cells were checked daily for signs of CPE and the whole culture was collected when 70% of the cells detached from the dish or showed granulation. This was followed by three cycles of freeze–thaw on dry ice and the collected P1 stock was titered using plaque assays. Using this stock, we inoculated 50 mL of fresh Sf9 suspended cell culture in polycarbonate Erlenmeyer flasks (Corning, Corning, NY, USA) at a density of 2.5 × 10^6^ cells/mL to create the P2 stock, which was harvested when ~70% of the cells were affected by CPE.

### 2.2. Purification of Virus-like Particles

The Sf9 culture was collected and centrifuged at 3000× *g* for 15 min, and the pelleted cells were disrupted by three cycles of freeze–thaw on dry ice. This lysed cell pellet was then resuspended in 1 mL of 1×TNTM pH8 (50 mM Tris pH8, 100 mM NaCl, 0.2% Triton X-100, and 2 mM MgCl_2_) and centrifuged again. The supernatant was mixed back with the cell culture supernatant and subjected to treatment with 250 units of benzonase nuclease (Sigma-Aldrich, Saint Louis, MO, USA) per 10 mL. The liquid was mixed with 1×TNET pH 8 (50 mM Tris pH 8, 100 mM NaCl, 0.2% Triton X-100, and 1 mM EDTA) in a 1:1 ratio and concentrated on a sucrose cushion of 20% sucrose in TNET, using a type 45 Ti rotor for 3 h at 4 °C and 203,500× *g* on a Beckman Coulter S class ultracentrifuge. The pellet was resuspended in 1 mL of 1×TNTM pH 8 and, after overnight incubation, purified on a 5 to 40% sucrose step gradient for 3 h at 4 °C and 209,500× *g* on the same instrument in an SW 41 Ti swinging bucket preparative ultracentrifuge rotor. Sucrose fractions known to correspond with small viral capsid buoyancy at the 10%, 15%, 20%, 25%, and 30% interfaces were aspired by a needle puncture and a 10 mL volume syringe. The purified fractions were dialyzed against 1× PBS phosphate-buffered saline (PBS; 137 mM NaCl, 2.7 mM KCl, 10 mM Na_2_HPO_4_, 1.8 mM KH_2_PO_4_) to remove the sucrose. Once the dialysis was complete, the fractions were subjected to thorough concentration on an Amicon Ultra-15 spin column (Sigma-Aldrich, Saint Louis, MO, USA) with a 100 kDa molecular weight cut-off.

### 2.3. Mass Spectrometry

For protein mass spectrometry, Coomassie-stained SDS-PAGE bands were fixed by methanol and acetic acid, excised with sterile scalpels, and placed in sterile ultrapure water. The bands were alkylated, trypsin-digested, extracted, and dried. Liquid chromatography–tandem mass spectrometry (LC-MS/MS) was conducted using a nano LC (Dionex Ultimate 3000 RLSCnano System, ThermoFisher, Waltham, MA, USA) interfaced with an Eclipse Tribrid mass spectrometer (ThermoFisher, Waltham, MA, USA). Data were searched using a local copy of Mascot v3.1 (Matrix Science, Mount Prospect, IL, USA). Mascot DAT files were parsed using Scaffold 5 (Proteome Software, Portland, OR, USA) for validation and filtering, and to create a non-redundant list per sample.

### 2.4. Preparation of cryoEM Grids and Plunge Freezing

UltrAuFoil R1.2/1.3 300-mesh grids were glow discharged for 300 s and the samples were plunge-frozen into liquid ethane using a Leica EM-GP plunge freezer (Leica Microsystems, Wetzlar, Germany) at 100% humidity and ambient temperature, applying front blotting only. The grids were clipped into autoloader grids and imaged using a Talos Arctica transmission electron microscope (TEM) (Thermo Fisher), equipped with a Gatan K2 direct electron detector, operated in low dose mode.

### 2.5. Collection of High-Resolution Data and 3D Reconstruction

Data collection parameters and refinement statistics are shown in Table 1. A 100 μm objective aperture was employed to truncate spatial frequencies beyond ~2.0 Å. The clipped CryoEM grids were imaged using the Talos Arctica electron microscope, operated at 200 kV, with a 5 s exposure and a total dose of 31 e^−^/Å^2^, divided into frames of 0.2 s. Movie frames were recorded in counting mode using the Serial EM suite [31] at a sampling of 1.038 Å/pixel. The dataset was processed in real time, using the cryoSPARC Live on-the-fly processing system of cryoSPARC 4 [32]. Micrograph quality was assessed by CTF estimation using a box size of 512. The subset of micrographs with the best CTF fit values were included in further processing. Particles were automatically boxed by the blob-picking subroutine of cryoSPARC Live. Boxed particles were curated by 2D classification in an iterative process, being repeated until a subset of particles only included clear 2D-class averages of the icosahedral particle. Ab initio model generation was carried out using the initial 100 particles, and the obtained model was used as a template in subsequent particle picking. The obtained initial volume was subjected to automatic refinement under icosahedral constraints and iterated refinement until pseudoconvergence. To improve the resolution, corrections for higher-order aberrations, beam tilt, trefoil, and anisotropic magnification were applied, as well as per-micrograph astigmatism and per-particle CTF parameters. To further improve the resolution, we used the non-uniform refinement subroutine of cryoSPARC 4 with a positive Ewald sphere curvature correction. The resolution of the reconstructed map was calculated based on a Fourier shell correlation (FSC) of 0.143.

The obtained cryoEM map was subjected to both de novo and sequence-specific model building using ModelAngelo [33]. In the latter case, all open reading frames (ORFs) were included to find potential sequence matches to the obtained electron density map. The final model was visualized and further refined in Coot [34], followed by another round of real space refinement in ISOLDE [35]. Visualization was carried out using UCSF Chimera [36] and Chimera X [37]. The final refinement step was carried out using PHENIX [38], refining a single subunit surrounded by the five other directly interacting subunits.

Analysis of the interface biophysical characteristics was carried out using PDBePISA v1.52 [39]. Homology modeling was performed using ColabFold v1.5.5, and AlphaFold2 with the MMseqs2 algorithm was used to generate query–template alignments [40,41]. The prediction was run in custom template mode, providing the SsChPV subunit structure as the template. This greatly improved the reliability of the prediction compared to the low (<60) overall plDDT scores and unreasonable folding of previous attempts without a custom template. The approximate inner volume of each capsid was measured in Chimera X [37] using the “measure volume” command. This operation estimates volumes slightly more conservatively than some older techniques [42], resulting in lower computed Matthews coefficients for AAV by 0.11Å^3^/Da.

## 3. Results

### 3.1. Protein Expression and Composition

**Figure 1:**
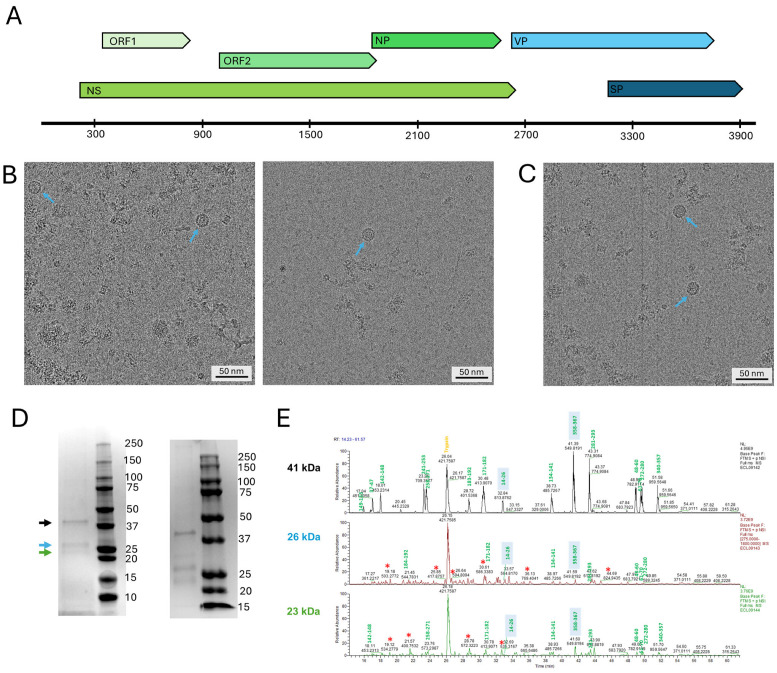
Analysis of the composition of the Syngnathus scovellis chapparvovirus (SsChPV) capsid protein. (**A**) Schematic diagram of the SsChPV genome; open reading frames (ORFs) of the non-structural (NS) expression cassette are marked by various shades of green, while ORFs located within the structural protein (VP) cassette are presented in shades of blue. Abbreviations: NP—nucleoprotein, SP—putative structural protein. The axis represents the genome position and is labeled in increments of nucleotides (nt). (**B**) Cryo-electron micrographs of particles expressed by the co-infection of SsChPV-VP-BAC and SsChPV-SP-BAC bacmids expressing the VP and SP proteins, respectively. (**C**) Cryo-electron micrograph of particles expressed by the SsChPV-VP-BAC only, encoding the VP protein exclusively. (**D**) SDS-PAGE analysis of the particles purified from the VP–SP suspension (left panel) and from the VP-only suspension (right panel). The ladders indicate the molecular weight of each fraction in kDa. The arrows show the protein bands excised and subjected to LC-MS/MS protein sequencing. (**E**) Results of the LC-MS/MS sequencing from panel (**D**). Peaks labeled by green numbers were identified as peptides of the VP ORF. The shaded background indicates peptides corresponding with the N- and C-termini of the ORF. Peaks marked by a red star are of host origin from the Sf9 moth cell line of *Spodoptera frugiperda*.

The 4001 nt long SsChPV genome, for which the terminal hairpin sequences have not been resolved, is unique among ChPVs as it contains two ORFs in its putative *cap* cassette (Figure 1A). The 367 aa long VP is a homolog of the single ORF present in the ChPV VP expression cassette [25,29,30], exhibiting 50–53% and 29–35% aa sequence identity to the corresponding predicted protein product in members of the *Ichthamaparvovirus* and *Chaphamaparvovirus* genera, respectively. The 235 aa long SP, however, is specific for the SsChPV genome and for a related EVE identified in the genome of another syngnathid fish, the tiger tail seahorse (*Hippocampus comes*) [30]. To express these proteins, we constructed three bacmids. SsChPV-whole-BAC included the complete coding genome cloned into the pFastBac dual promoter-knocked-out backbone, whereas SsChPV-VP-BAC and SsChPV-SP-BAC included only the VP or SP coding sequence, respectively, linked to the polyhedrin (Pph) promoter of the pFastBac1 expression vector. Sf9 cultures were infected by the P1 stocks of each bacmid construct separately. In parallel, Sf9 cultures were infected with combinations of SsChPV-VP-BAC and SsChPV-SP-BAC at supernatant ratios of 1:3, 2:2, and 3:1. The density gradient purification did not yield any observable bands that would suggest the presence of virus-like particles (VLPs). We pooled all fractions that may correspond to the buoyancy of PV capsids or VLPs. These were concentrated 200× more than their post-dialysis volume. There were no particles present in the SsChPV-whole-BAC and SsChPV-SP-BAC construct preps upon screening by cryo-EM. Both the SsChPV-VP-BAC-concentrated suspension and the mixed preparation of SsChPV-VP-BAC and SsChPV-SP-BAC contained a low concentration of assembled VLPs of approximately 21 nm in diameter (Figure 1B,C). Both suspensions were composed of the same protein fractions upon SDS-PAGE analysis (Figure 1D), running at 41 kDa, 26 kDa, and 23 kDa, respectively. Protein sequencing by LC-MS/MS revealed that only the 41 kDa band was derived from the SsChPV genome, composed entirely of the VP product, corresponding with the full length of the VP ORF. This indicates that VP runs at its predicted molecular weight of 41 kDa and is translated from the first ATG start codon of its frame. Both smaller bands were composed of fragments of VP, probably as the result of capsid degradation, as well as of host proteins from the fall army moth (*Spodoptera frugiperda*) cell line. There were no peaks of host protein or baculoviral contamination in the 41 kDa band.

### 3.2. Structural Studies

Particles purified from the mixed SsChPV-VP-BAC+SsChPV-SP-BAC suspension were vitrified and imaged to resolve their 3D capsid structure using cryo-EM. Out of the 7182 micrographs that were incorporated in the reconstruction, approximately one in four micrographs contained a particle, suggesting a very low assembly rate. In spite of the low particle count, we obtained the SsChPV capsid structure at a resolution of 2.93 Å. The density map and corresponding atomic model were deposited into the EMData Resource and the PDB under the accession numbers EMD-49314 and 9NEK, respectively.

**Figure 2:**
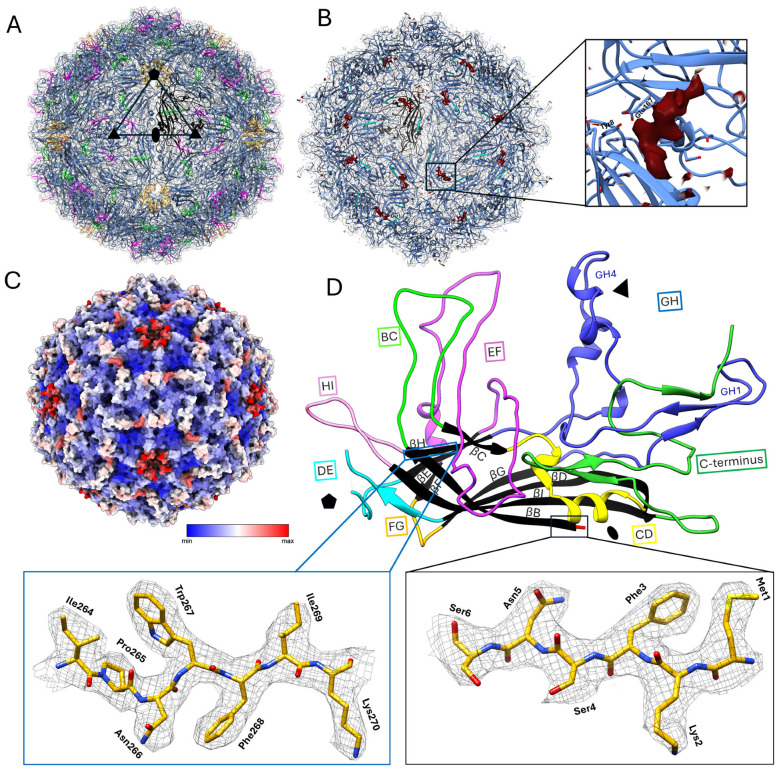
3D reconstruction and structural analysis of the Syngnathus scovelli chapparvovirus (SsChPV) capsid. (**A**) Reconstruction showing the SsChPV particle surface as the transparent electron density, overlaying the atomic model of the complete 60 mer icosahedral assembly, which is represented as ribbon diagrams. A single subunit is highlighted in black. Regions highlighted in green correspond with the poorly ordered surface-exposed region of the EF loop. Yellow indicates the poorly ordered segment of the DE loop, with its apex being completely disordered in the Coulomb potential density. The last five ordered residues of the C-terminus are highlighted in magenta and are also surface-exposed. The last ten residues of the C-terminus are also disordered and absent. A single asymmetric unit of the *T* = 1 icosahedron is indicated by the triangle, with a pentagon marking the fivefold symmetry axes, an ellipsoid the twofold symmetry axes, and triangles the threefold symmetry axes. (**B**) Cross-section view of the SsChPV particle, exposing the capsid lumen. A single subunit is highlighted in black. The first five N-terminal residues that also coincide with the first five residues of the structural protein-encoding open reading frame are indicated in cyan. The unmodeled piece of density is highlighted in red. The magnified panel displays the pocket, which is occupied by the small, unidentified molecule. (**C**) Surface view of the SsChPV atomic capsid model, colored by the average *B*-factor values associated with each residue. (**D**) Ribbon diagram representation of a single subunit of the SsChPV capsid. The conserved β-strands of the eight-stranded jelly roll core are shown in black. Each loop is colored separately, as indicated by the text box frames. Symmetry axes are marked with the same symbols as in panel (**A**) for orientation. The left inset shows a representative example of the atomic model vs. the density fit, while the right inset shows the ordered density of the first five N-terminal residues, starting with Met1.

The SsChPV capsid exhibits *T* = 1 icosahedral symmetry and is assembled from 60 copies of a single VP (Figure 2A). Although the SsChPV capsid is among the smallest PV capsids at 22 nm at its largest dimension, the inner capsid volume of 2.179 × 10^6^ Å^3^ exceeds the volume of the PmMDV particle, despite comparable genome sizes (Table 2). With a Matthews coefficient (*V*_m_) comparable to AAV and significantly greater than PmMDV, SsChPV has no need to compress its genome any more than the typical parvovirus.

The overall smooth capsid surface displayed two openings: an opening at the fivefold axis of symmetry and an opening at the threefold axis. Although the capsid lumen was expected to be empty because the self-assembled PV VLP was expressed in the absence of viral genomic material, a density that did not correspond to the VP protein could still be detected (Figure 2B). The shape of this density is consistent with a polypeptide of at least seven amino acids in length and is not consistent with a nucleic acid, although it is conceivable that the density could instead represent a small molecule or the superposition of more than one molecule. Neither the VP nor the SP protein sequence could be assigned to the density using ModelAngelo [33]. This density of unknown identity is non-covalently bound to the capsid interior, occupying a pocket comprising negatively charged residues within the hydrophobic interior wall (Figure 2B).

We could successfully build an atomic model of the electron density, encompassing almost the entire icosahedral shell except for six unmodeled residues at the peak of the poorly ordered DE loop, located above the fivefold opening (Figure 2A). Another poorly ordered region of 7 aa (VP residue range of 112–119), which could only be visualized at an isosurface threshold of 2σ, was located within the EF loop and is surface-exposed (Figure 2A). Both regions are associated with increased flexibility, confirmed by the high *B*-factor of their atoms (Figure 2C). The SsChPV capsid structure also displays the canonical eight-stranded jelly roll core and long linking loops that are typical of the *Parvoviridae* (Figure 2D). The ordered range encompassed 351 residues, including all residues except the DE loop peak and the 10 C-terminal residues. The C-terminus is exposed to the surface near the opening at the threefold symmetry axis (Figure 2A). All other PV capsid structures have a disordered N-terminus, but SsChPV is ordered from the first residue, which is the translation start site of the VP ORF (Figure 2D). Compared to other PV capsids, SsChPV lacks N-terminal elements such as the βA strand; Met1 is part of the βΒ strand (Figure 2A,D). Each subunit exhibits a rather short GH loop composed of only two subloops, namely GH1 and GH4, as opposed to the three or more subloops typically found in this location. The SsChPV capsid subunits also possess a long, mostly surface-exposed C-terminal segment, as opposed to the buried C-termini of the *Parvovirinae*.

### 3.3. Structure Comparison and Multimer Interactions

To analyze the SsChPV capsid structure in the context of other virus capsids, we subjected a single subunit to structural comparison using the DALI server [43] (Table 3). SsChPV was more structurally similar to viruses of the *Parvovirinae* subfamily (such as AAV) than to members of its own subfamily, with *Z*-scores of 16.8–18.4. Scores for pairwise comparisons of SsChPV to fellow members of the *Hamaparvovirinae* were worse (at 11.3–11.8) than to members of any other parvovirus subfamily, even though SsChPV and other *Hamaparvovirinae* have in common a smaller capsid diameter than the *Parvovirinae* and *Densovirinae* subfamilies. Non-parvovirus structures yielded significantly lower scores (≤5.6).

**Figure 3:**
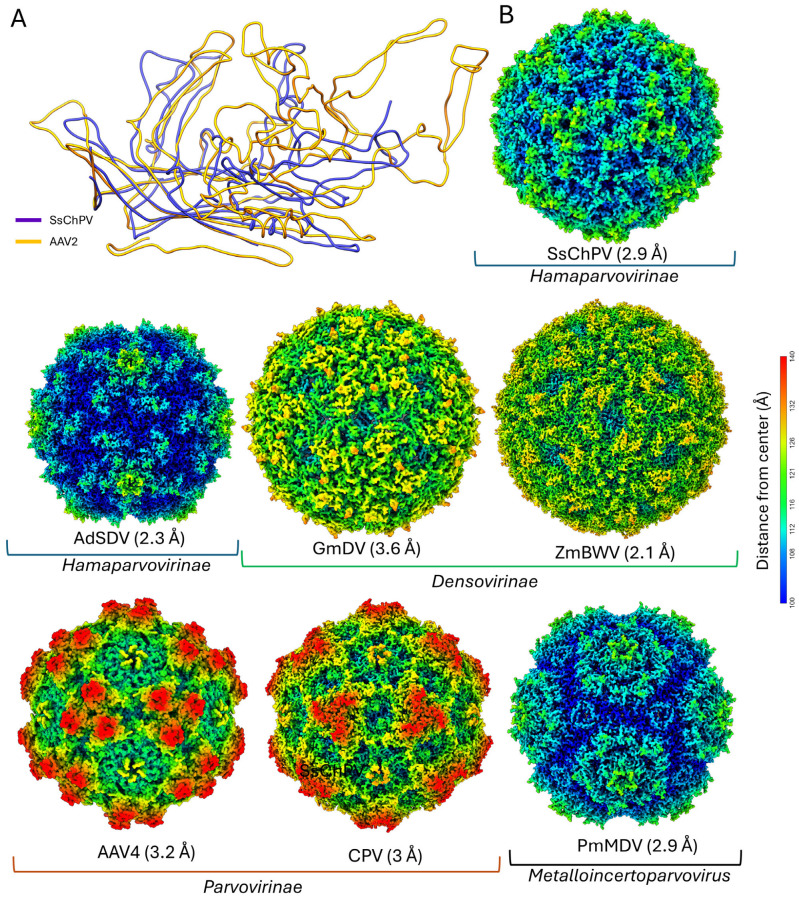
The Syngnathus scovelli chapparvovirus (SsChPV) capsid in a structural context with other parvoviruses. (**A**) Superimposed ribbon diagrams of the SsChPV subunit and adeno-associated virus 4 (AAV4) of PDB ID 2G8G, scoring the highest structural similarity scores as measured with the DALI structure comparison server. (**B**) Comparison of the SsChPV capsid surface to representatives of all major parvoviral lineages. The surface diagrams are radially colored from the capsid center on a uniform scale. The icosahedral particles are oriented by the I1 convention, with the twofold symmetry axis facing the viewer in the *z*-plane, while the threefold axes are in the *x*-plane, and the fivefold axes are in the *y*-plane. This orientation is identical to that of panel A in Figure 2. Abbreviations: AdSDV—Acheta domesticus segmented densovirus, AAV4—adeno-associated virus 4, CPV—canine parvovirus, GmDV—Galleria mellonella densovirus, PmMDV—Penaeus monodon metallodensovirus, ZmBWV—Zophobas morio black wasting virus.

The superposition of the SsChPV VP monomer onto AAV4 shows structural analogies between the GH loop structures of AAV and the C-terminal loops of SsChPV (Figure 3A). In addition, both viruses have extended BC and EF loops, a characteristic not shared with evolutionary intermediates between them. Although these homoplasies are not the reason that SsChPV matches AAV4 better than other hamaparvoviruses (as deleting them from the DALI search template does not change which subfamily matches the best), the parallelism is of interest. When the SsChPV capsid surface is visually compared to other PV representatives, the dissimilarity becomes even more apparent (Figure 3B). The SsChPV capsid surface exhibits a deep depression at the twofold symmetry axis and small spikes surrounding the threefold axis, responsible for its unique surface morphology, setting the SsChPV capsid clearly apart from the rest of the family.

**Figure 4:**
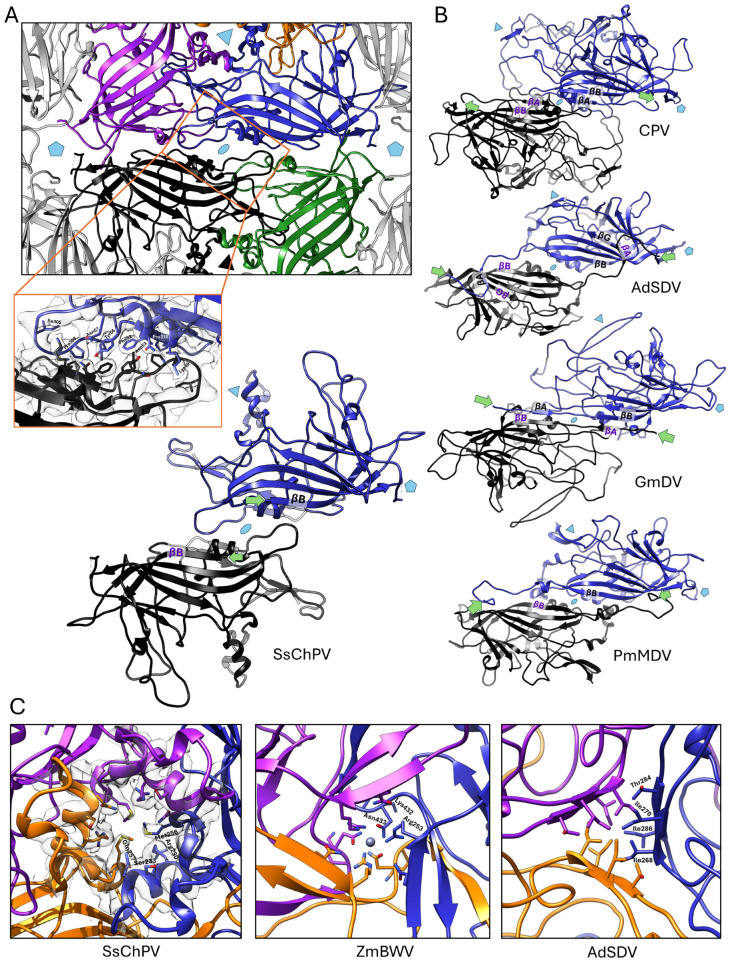
Multimer interactions at the Syngnathus scovelli chapparvovirus (SsChPV) twofold and threefold symmetry axes. (**A**) The SsChPV twofold axis in context with its neighboring subunits from the capsid interior. The blue and black ribbon diagrams indicate the dimer, while the magenta and gold diagrams represent the threefold subunit and the green diagram shows the fivefold neighboring subunit to the monomer highlighted in blue. Pentamers mark the fivefold symmetry axes, triangles the threefold symmetry axes, and the ellipsoid the twofold symmetry axes. The magnified panel shows the sidechains with corresponding electron density, which are responsible for keeping the dimer together. (**B**) Comparison of the SsChPV’s dimer architecture with all the twofold interface-forming strategies of parvoviruses thus far. The two subunits are represented by the black and blue ribbon diagrams, respectively. The first ordered N-terminal residue of each subunit is marked by the green arrows. The interacting β-strands are labeled accordingly. Canine parvovirus (CPV) represents the *Parvovirinae* subfamily, where βA–βB strand interactions occur within the same subunit. Acheta domesticus parvovirus (AdSDV) of the *Hamaparvovirinae* displays the domain-swapping conformation, which facilitates the interaction between the βA and βG of the two distinct subunits. Galleria mellonella densovirus (GmDV) of the *Densovirinae* displays the canonical domain-swapping conformation of βA–βB interactions. Penaeus monodon metallodensovirus (PmMDV) is of a divergent lineage without a subfamily affiliation and constructs its capsid interior of βB–βB interactions in a domain-swapping conformation. (**C**) Comparison of the pore at the SsChPV threefold symmetry axis with the β-annulus-like threefold pores of a densovirus, Zophobas morio black wasting virus (ZmBWV), and AdSDV, a hamaparvovirus. While the pores of SsChPV and ZmBWV are similarly sized at 13 Å and 12 Å, respectively, the ZmBWV β-annulus coordinates an ion. The 10 Å wide pore in AdSDV relies on hydrophobic interactions for stability. Each subunit comprising the trimer is represented by a different color.

Upon examining the interior surface of the SsChPV capsid, it becomes apparent that the depression near the twofold symmetry axis is due to the lack of interaction between the two subunit backbones (Figure 4A). We calculated the size of the area buried due to interacting atoms at each interface, as well as the amount of free energy gained when they form (Table 4). The area enclosed by the twofold symmetry interface is significantly smaller than that of both other interfaces at 751.9 Å^2^, gaining only −8 kcal/mol of solvation free energy as the hydrophobic interface forms.

In PV structures, the twofold symmetry axis is typically supported by direct interactions between the β-strands of the jelly roll core (Figure 4B), but SsChPV’s β-sheets are too far apart to interact. Measured at the Cα atoms of Met1, the distance between the neighboring βB strands is 19.1 Å, as opposed to only 7.3 Å in the case of PmMDV, harboring direct twofold βB–βB interactions (Figure 2B). The extensive gap at the SsChPV twofold axis is occupied by the bulky sidechains of aromatic and large hydrophobic residues, which create an expansive hydrophobic interface (Figure 4A). None of these sidechains, however, are donated by the βB, but rather by the conserved αA helix and CD loop (Lys49, Phe 52, Tyr56, and Asn57), by the EF loop (Ile105), or by the long C-terminal segment (Pro308 and Pro 316).

The threefold symmetry axis in the SsChPV capsid creates an hourglass-shaped invagination; the pore present in homologous capsids is closed in this capsid only around Met256 and varies in diameter from 5 Å–13 Å above and below that site (Figure 4C). The wall of this opening comprises three α-helices, donated by the GH4 subloop of each subunit. The pore is lined by residues of large sidechains, i.e., Arg230, Met256, and Gln257. Only the Met256 sidechain is oriented toward the pore, with the positively charged NE and NH atoms of Arg230 facing away from the opening, resulting in a uniformly hydrophobic surface to line this threefold pore.

**Figure 5:**
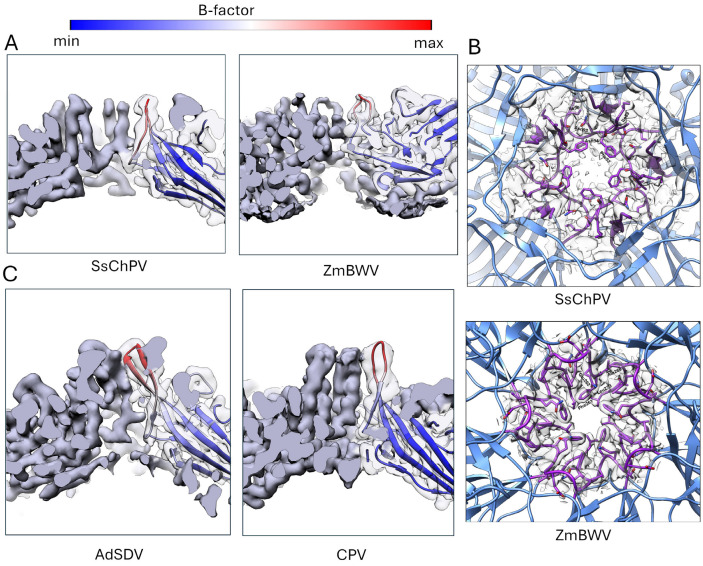
Comparison of the Syngnathus scovelli chapparvovirus (SsChPV) fivefold-related multimer interactions with those of other parvoviruses. (**A**) Cross-section view of the short, degraded channel at the SsChPV fivefold symmetry axis (left panel), with it facing outward from the fivefold pore. Only members of the *Densovirinae* subfamily, represented here by the Zophobas morio black wasting virus (ZmBWV), display a similarly reduced fivefold channel (right panel). The electron density map of a single subunit of the pentamer is shown as transparent, revealing the atomic model as a ribbon diagram, colored by its B-factor. Both density maps were filtered to a resolution of 3.5 Å to minimize noise. (**B**) The top–down view of the SsChPV fivefold symmetry axis shows the lack of ordering in the residue sidechains associated with the DE loop (purple), unlike the well-defined sidechain densities of corresponding residues lining the ZmBWV fivefold channel (bottom panel). (**C**) The typical, well-defined fivefold channels of the *Hamaparvovirinae* and the *Parvovirinae*, to compare with those seen in panel (**A**). The panel follows the color and representation scheme of panel (**A**). Abbreviations: AdSDV—Acheta domesticus parvovirus, CPV—canine parvovirus.

The SsChPV fivefold symmetry axis also opens in a pore, similar to other PVs (Figure 5A). The opening is occupied by the sidechains of the hydrophobic Phe92 and the hydrophilic Asn94, both of which lack ordered sidechain density. The presence of large quantities of discontiguous, disordered density within the pore may originate from the sidechains of these residues, which are probably mobile (Figure 5B). This is in concordance with the high B-factor assigned to the entire fivefold region (Figure 2C). Typically, the fivefold pore continues in a well-defined channel, formed by β-strands delegated by the DE loop, with increased flexibility limited to the loop peak (Figure 5C). In SsChPV, however, the ascending and descending stems of the DE loop comprise flexible coils instead of β-strands, which prevent the formation of a similarly long, more rigid channel wall (Figure 5B).

### 3.4. Structural Predictions of Further Chapparvoviral Structures Through the Syngnathus Scovelli Chapparvovirus Capsid

**Figure 6:**
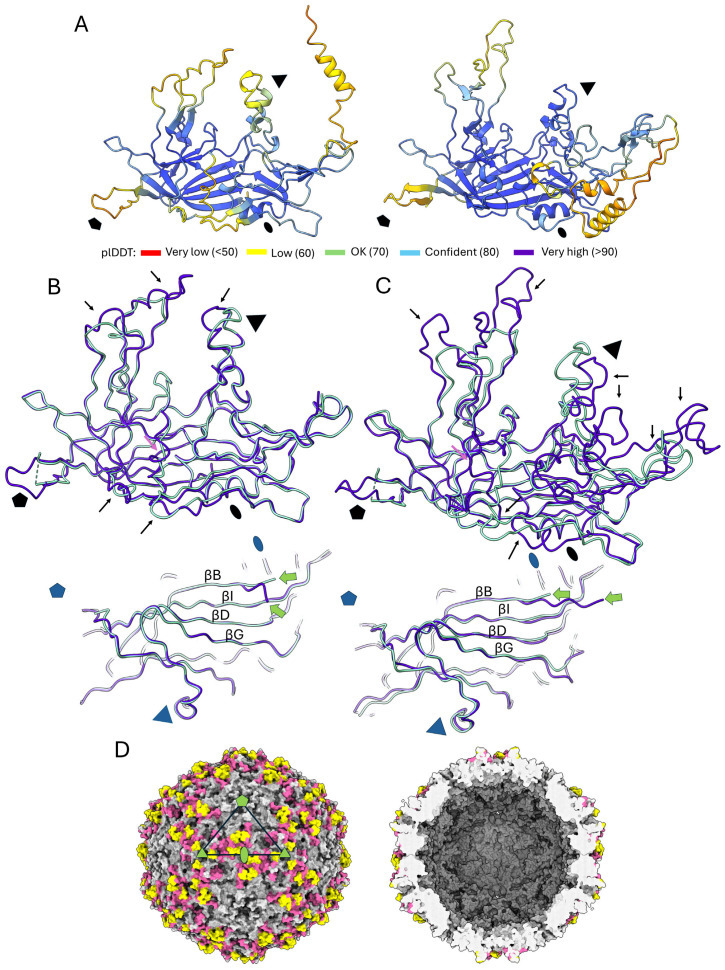
Examining the structural evolution of the chapparvovirus (ChPV) capsid using SsChPV as a homology guide in custom template mode. (**A**) Homology models of a single subunit of two representative chapparvoviruses, the ichthamaparvovirus Neolamprologus niger chapparvovirus (NnChPV) (left) and the chaphamaparvovirus mouse kidney parvovirus (MKPV), shown as ribbon diagrams. Both models are colored according to the plDDT scores color scheme of AlphaFold2, with higher scores indicating higher confidence in the structural predictions. (**B**) Superimposition of the SsChPV subunit structure (pale green ribbons) with the homology model of NnChPV (purple ribbons). The top panel shows the side view of the subunits, while the bottom panel displays them from a view of the capsid interior. The green arrows point at Met1 of the structural protein encoding the open reading frames of both viruses. Regions accommodating insertions are indicated by black arrows, while a pink arrow points to the flexible region of the EF loop, which is conserved among other parvoviruses. The fivefold symmetry axis is marked by a pentagon, and the twofold and threefold symmetry axes are indicated by an ellipsoid and a triangle, respectively. (**C**) Superimposition of the SsChPV subunit structure (pale green) with the homology model of MKPV (purple). The panel is colored and marked as described in panel (**B**). (**D**) Regions of insertions and possible distinct confirmation in MKPV vs. SsChPV (magenta) and in NnChPV vs. SsChPV (yellow), shown mapped to the SsChPV capsid surface. The left panel displays the capsid surface, while the right panel shows the capsid interior. An asymmetric unit is highlighted by the triangle, with symmetry axes represented by the same shapes as in panels (**A**,**B**).

As the SsChPV capsid structure is the first one from the ChPV lineage to have been resolved, we aimed to investigate which aspects of its structure are type-specific or conserved throughout the ChPV clade. To this end, we used AlphaFold2 [40] to construct homology models based on the SsChPV structure, taking advantage of the high sequence similarity among ChPVs. The genus *Ichthamaparvovirus*, which contains SsChPV, was represented by Neolamprologus niger chapparvovirus (NnChPV). NnChPV was derived from the metagenomic assembly of a Lake Tanganyika cichlid, Neolamprologus niger [44]. To represent the *Chaphamaparvovirus* genus, we selected the MKPV. The NnChPV VP-derived protein sequence shares 53.21% aa identity with the SsChPV VP, whereas this similarity is 30% against MKPV. Overall, predictions of high confidence could be generated, with plDDT scores of over 90 throughout almost the entire subunit (Figure 6A,B). Lower confidence scores of ~60 were associated with the peaks of surface-exposed loops, such as the BC, EF, and GH4 loops and the C-terminal segment. As the DE loop peak is absent from the SsChPV structure, the conformation of the corresponding section could not be predicted confidently.

All predictions indicated Met1 to be the first ordered residue of both homology models, located in a very similar position to the SsChPV Met1, i.e., at the beginning of the βB strand (Figure 6A,B). Although the peak of the GH4 loop was predicted to adopt a conformation that diverges from that of the SsChPV, the α-helix comprising the wall of the threefold pore was predicted to be conserved in both NnChPV and MKPV. Both homology models exhibited the long C-terminal segment, which was predicted to adopt a similar confirmation in NnChPV to that of SsChPV despite a small 4 aa long insertion present near the twofold axis (Figure 6A). The same segment of MKPV is significantly longer at 121 aa vs. only 65 residues in SsChPV, which is due to several insertions. Despite this, the MKPV model also harbors the loop at the twofold axis, which contributes to assembling the twofold interface (Figure 6B).

In addition to the peak of the EF loop, which contains a large insertion in both models, the flat surface-exposed region, which is highly flexible and poorly ordered in SsChPV, may also accommodate insertions. When mapping all regions affected by these insertions to the SsChPV capsid map, they concentrate at the twofold depression and around the threefold symmetry axis (Figure 6C). Insertions were not found to affect the interior wall of the capsid or the buried threefold interfaces.

## 4. Discussion

We successfully determined the capsid structure of a ChPV, which is also the first PV capsid ever studied to infect a fish host. The structural analysis of ChPVs has proven to be challenging previously. A typical PV infection is associated with intranuclear inclusion bodies of numerous icosahedral particles, facilitating a diagnosis by thin sectioning and transmission electron microscopy (TEM) (e.g., [45,46,47,48]). This is not true for ChPVs; despite ultramicroscopic examinations in mice with ChPV-induced tubular nephropathy, no intact particles could be observed within inclusion bodies or elsewhere in infected tissue [25,29]. We could recombinantly express and purify intact ChPV particles, but only at very low concentrations. This is also unusual for a PV, as self-assembling PV VLPs can be routinely expressed and purified in large quantities using a baculovirus expression system [7,49,50,51].

The position of the ORF of the SP within the genome and its proposed origin would suggest a structural function [30]. Our results, however, show that the SP product is not incorporated into the SsChPV capsid. Although the unmodeled density near the fivefold axis may display a protein-like morphology, our MS/MS and modeling results exclude the possibility of it being derived from SP. Small molecules of non-viral origin, binding in pockets of similar disposition, are termed pocket factors and have been shown to play important roles in the particle assembly of hepadnaviruses [52], picornaviruses [53], and flaviviruses [54]. A similar function may be associated with this small molecule of unknown identity. As the capsid only comprises the full-length product of the VP, the SP is probably expressed as a late non-structural protein. The SsChPV and two shrimp-infecting PVs (PstDV and PmMDV) [7,55] use a single VP each to assemble their capsids, while other characterized parvoviruses form mosaic capsids from multiple isoforms of their VP.

Despite being a vertebrate-infecting PV, the capsid surface morphology and architecture of the SsChPV are distinct from those of the widely investigated *Parvovirinae*, which is in concordance with the phylogenetic position of the ChPV clade. The ChPV capsid structure is also an outlier among the structurally characterized *Hamaparvovirinae* members, making the lineage structurally divergent. The highest DALI scores, however, were with members of the *Parvovirinae* as opposed to the *Hamaparvovirinae*. Using the experimentally determined structure and homology models, we could show that the apex of the surface loops are locations of insertions in the mammalian ChPV, MKPV. This is analogous to the evolution of the *Parvovirinae* surface spikes, which are significantly shorter and blunter in hosts with a less prominent adaptive immune system, such as reptiles [56,57,58]. In addition to the surface spikes, a smooth but flexible segment of the EF loop also proved to be a variable region. The corresponding segment, however, is structurally conserved in all three subfamilies thus far [5]. Both the polymorphism and the flexible disposition imply that this region could play an important role in host–virus interactions, such as receptor attachment.

The presence of long, disordered N-terminal VP domains has been a consistent characteristic of the *Parvoviridae*, regardless of taxonomic affiliation [5]. The flexibility of these domains is necessary to perform the conformational changes required to externalize enzymatic regions, membrane-penetrating peptides, or nuclear import and export signals through the fivefold channel [5,7,10,14,59,60]. These domains are often required for endosomal egress during trafficking to the intranuclear replication site [7,61]. As the Met1 translation start of the SsChPV VP also coincides with being the first residue of the conserved eight-stranded jelly roll core, the presence of any subsequent N-terminal domains can be excluded. This is a trait that appears to be conserved throughout both genera of the ChPV lineage. Although some invertebrate-infecting PVs of the *Densovirinae* subfamily attach long N-terminal domains to their major capsid protein-encoding ORFs with alternative splicing [62,63], transcriptome studies of the MKPV and capuchin kidney parvovirus did not find any evidence of this in ChPVs [25,29], neither did the *in silico* analysis of SsChPV’s transcription strategy [30].

Unlike in most PVs thus far, the SsChPV fivefold pore is not surrounded by a channel-like structure due to the DE loop being disordered and facing away from the pore. The shortened and less-defined disposition of the channel has been found in two independent PV lineages thus far: the invertebrate-infecting *Densovirinae* subfamily and human parvovirus B19 (B19V) of the *Parvovirinae* [51,63,64,65,66]. Both lineages, however, were shown to have permanently externalized minor capsid protein N-termini, as opposed to the rest of the family, where the externalization is dependent on pH or genome packaging [7,10,11,12,14]. Even in these PVs, however, the fivefold channel is lined by large hydrophobic residues (typically phenylalanine) to provide a hydrophobic surface for the N-terminus to slide on [5]. The SsChPV fivefold pore also harbors the phenylalanine ring, which is conserved in both the NnChPV and MKPV. We hypothesize that the degeneration of the channel happened in response to the absence of the N-terminal domains, rendering one of its functions obsolete. The presence of a large, 15 Å wide pore, however, still suggests that the fivefold symmetry axis may be the location of genome packaging and uncoating. These processes may require the hydrophobicity provided by the phenylalanine sidechains. Alternatively, this hydrophobicity might be required to stabilize the interface, even if the pore itself has lost its original function.

In addition to mobile functional domains, the VP N-termini also facilitate interactions between the β-strands at the twofold symmetry axis. Within the *Parvovirinae*, the N-terminus adopts a hairpin-like loop and interacts with the βB strand of the same subunit, leaving a relatively weak twofold interface. In the rest of the family, however, the N-terminus interacts with the twofold neighboring subunit, adopting a domain-swapped conformation [5,7,11]. This not only results in a strong twofold interface but also creates inter-subunit β-sheets, which can extend the lumen and the packaging capacity of the capsid [63,65]. The SsChPV capsid structure, however, adopts neither, resulting in an even weaker twofold interface. As both the absence of the N-terminus and the presence of the twofold-extended EF loop and C-terminus appear to be conserved throughout the ChPV lineage, it is likely that ChPVs share aspects of their intracellular trafficking, which requires quick particle disassembly, e.g., during uncoating. The presence of degraded VPs and VP fragments, along with the extremely low number of intact capsids, suggests low particle stability, which may be the reason why no ChPV particles have been seen or isolated thus far in vivo.

The parvoviral threefold axis is divergent throughout the family; while the *Parvovirinae* unanimously exhibit a closed threefold axis, the rest of the family has been found to possess a pore-like opening at this axis as well [5]. These pores are formed by a β-annulus-like structure, as their walls are assembled of three or more β-strands, which interact across the pore by hydrophobic interactions, hydrogen bonds, or by coordinating an ion [7,11,51,55,63,64,65]. Larger β-annuli of both the *Parvoviridae* and the *Tombusviridae* were found to rely on coordinating ions for stability as opposed to the other strategies [7,63,67]. As the β-annuli occur in a wide array of only distantly related PVs of non-homologous VP sequences, these structures have possibly evolved convergently [7]. Unlike the pores of a β-annulus-like architecture, the wall of the threefold depression in SsChPV is composed of three α-helices. Consequently, the β-annuli and the SsChPV threefold axis may harbor different evolutionary origins but structural and functional similarities.

The threefold axis is lined by the bulky sidechains of the methionine and arginine residues, yet only the three Met256 sidechains interact with each other. The methionine sidechain is hydrophobic yet very flexible, with an ability to transfer this flexibility to other residues through the reversible oxidation of the thioether group [68]. Consequently, methionine makes solvent-exposed surfaces ductile [68,69]. Heiby et al. [69] have shown that six core methionine residues lead the time-sensitive oligomerization process of spider silk spidroins, which are required to oligomerize at the very moment they pass through the spinning duct; otherwise, defective silk is produced. The sidechains of Met256 may play a similar role in the initial oligomerization of the SsChPV capsid trimers, which may be the pre-formed building blocks necessary to assemble the 60 mer. Hitherto, assembly has only been studied in the *Parvovirinae* subfamily, and all members of that subfamily lack a threefold pore. Despite this, the minute virus of mice (MVM) virion harbors bulky hydrophobic residues at the buried threefold interface. These residues were shown to be essential for assembly, while assembled trimers have been observed to accumulate in the cytoplasm of MVM-infected cells [70,71]. In contrast, trimer formation was found to be non-essential for a dependoparvovirus, AAV2, as opposed to the disruptive effects of mutating the twofold or the fivefold interfaces [72]. Met256, however, is not conserved in the closely related NnChPV or in the MKPV capsids, while the α-helices forming the wall of the pore appear to be. Met256 is substituted by phenylalanine in NnChPV and by glutamic acid in MKPV, both of which can establish strong, non-covalent connections via hydrophobic interactions or hydrogen bonds, respectively. This implies that trimer-based assembly might be conserved among ChPV capsids, although its biophysical mechanisms and dynamics may vary.

The function of the threefold-related pores of PVs has not been characterized. Initially, when the 10 Å wide pore of the Galleria mellonella densovirus was discovered, it was hypothesized to serve as a portal for genome packaging [65]. The frequent presence of ions and the overall rigidity of these structures, however, appear to contradict this [7,63]. In the case of a related densovirus, the Zophobas morio black wasting virus (ZmBWV), the partial removal of such an annulus-related ion was carried out, resulting in an increased number of degraded viral particles, which leaked the viral genome [63]. As the capsid could not remain intact in the absence of such ions, it is unlikely that these openings would be functional as portals. Moreover, similar β-annuli trimers frequently serve as the basic assembly units of self-assembling nanocages in polar solvents [73,74]. It is possible that these structures and their ChPV analogs solely assume an assembly function.

## 5. Conclusions

Despite the challenges of studying ChPV capsids, we could express, purify, and structurally characterize a chapparvovirus capsid for the first time. This provided the opportunity to not only analyze the SsChPV structure but also gain insights into the conserved and variable elements of ChPV structures. The capsid of SsChPV, which is small in diameter and comprises a single VP isoform, lacks the functional and structural contributions typical of a parvoviral N-terminus. It also does not possess the environmental stability and resilience that typifies the family. ChPVs appear to have evolved long surface loops independently from the other vertebrate-infecting lineage, the *Parvovirinae*, despite fundamentally different subunit structures and multimer interactions. The presence of an analogous structure to the β-annuli of denso- and hamaparvoviruses suggests that multiple PV lineages may utilize the same assembly strategy, relying on trimers as the basic assembly unit. Lastly, we hypothesized that the DE loop’s morphology and the degradation of the fivefold channel in SsChPV is an evolutionary response to the absence of the N-terminal domain. Taken together, ChPVs not only comprise an evolutionarily divergent PV lineage but also appear to possess a fundamentally distinct viral life cycle from those of other members within the family.

## Figures and Tables

**Table 1:** Processing and refining parameters of the data collection and image reconstruction of the Syngnathus scovelli chapparvovirus.

Processing and Refinement Parameters	
Total number of micrographs	11,904
Number of micrographs involved in reconstruction	7182
Reconstruction software	CryoSparc v4.5.3
Defocus range (µm)	0.6–2.4
Electron dose (e^−^/Å^2^)	31
Frames/micrograph	30
Pixel size at acquisition (Å/pixel)	1.038
Calibrated pixel size (Å/pixel)	1.011
Starting number of particles	14,213
Particles used for final map	2202
Resolution of final map (Å)	2.93
PDB ID	9NEK
Residue range (VP2)	1–356
Map correlation coefficient	0.8339
RMSD (root-mean-square deviation) [bonds] (Å)	0.004
RMSD [angles] (Å)	0.554
All-atom clash score	6.74
Favored (%)	95.46
Allowed (%)	4.54
Outliers (%)	0.00
Rotamer outliers (%)	0
C-β deviations	0

**Table 2:** Direct comparison of the genome size and approximate volume of parvoviral particles with similar genome sizes and experimentally resolved structures. Abbreviations: SsChPV—Syngnathus scovelli parvovirus, AdSDV—Acheta domesticus segmented densovirus, PstDV—Penaeus stylirostris densovirus, PmMDV—Penaeus monodon metallodensovirus, AAV2—adeno-associated virus 2.

	Inner volume (Å^3^)	Genome Size (nt)	Genus	Subfamily	*V*_m_ (Å^3^/Da)
SsChPV	2.179 × 10^6^	4002 *	*Ichthamaparvovirus*	*Hamaparvovirinae*	1.67
AdSDV	1.863 × 10^6^	3332/3316	*Brevihamaparvovirus*	*Hamaparvovirinae*	1.71
PstDV	2.143 × 10^6^	3914	*Penstylhamaparvovirus*	*Hamaparvovirinae*	1.67
PmMDV	2.059 × 10^6^	4371	*Incertometalloparvovirus*	Not assigned	1.44
AAV2	2.518 × 10^6^	4679	*Dependoparvovirus*	*Parvovirinae*	1.65
AAV6	2.7 × 10^6 †^	4683	*Dependoparvovirus*	*Parvovirinae*	1.76 ^†^

* Excluding terminal hairpins; ^†^ Measured by Speir and Johnson (2012) [42] from the crystal structure by alternate methods.

**Table 3:** DALI scores comparing the structure of the Syngnathus scovelli chapparvovirus capsid to other protein structures deposited in the RCSB Protein Data Bank.

Z-Scores	PDB ID	Identity %	Genus	Subfamily	Family
18.4	2g8g	13	*Dependoparvovirus*	*Parvovirinae*	*Parvoviridae*
18.2	3ntt	14	*Dependoparvovirus*	*Parvovirinae*	*Parvoviridae*
18.1	4rso	14	*Dependoparvovirus*	*Parvovirinae*	*Parvoviridae*
18.1	4qc8	10	*Dependoparvovirus*	*Parvovirinae*	*Parvoviridae*
18	4g0r	11	*Dependoparvovirus*	*Parvovirinae*	*Parvoviridae*
17.8	1mvm	11	*Protoparvovirus*	*Parvovirinae*	*Parvoviridae*
17.8	9c27	12	*Erythroparvovirus*	*Parvovirinae*	*Parvoviridae*
17.7	4dpv	10	*Protoparvovirus*	*Parvovirinae*	*Parvoviridae*
17.6	7kfr	14	*Dependoparvovirus*	*Parvovirinae*	*Parvoviridae*
17.6	6nf9	13	*Protoparvovirus*	*Parvovirinae*	*Parvoviridae*
17.5	7jot	14	*Dependoparvovirus*	*Parvovirinae*	*Parvoviridae*
17.5	4iov	13	*Dependoparvovirus*	*Parvovirinae*	*Parvoviridae*
17	7wq	14	*Dependoparvovirus*	*Parvovirinae*	*Parvoviridae*
17.4	2qa0	14	*Dependoparvovirus*	*Parvovirinae*	*Parvoviridae*
17.3	3ng9	14	*Dependoparvovirus*	*Parvovirinae*	*Parvoviridae*
17.1	1k3v	13	*Protoparvovirus*	*Parvovirinae*	*Parvoviridae*
17.1	3kic	14	*Dependoparvovirus*	*Parvovirinae*	*Parvoviridae*
16.8	1s58	12	*Erythroparvovirus*	*Parvovirinae*	*Parvoviridae*
15.2	3p0s	10	*Iteradensovirus*	*Densovirinae*	*Parvoviridae*
13.1	8t9c	9	*Blattambidensovirus*	*Densovirinae*	*Parvoviridae*
13	1dnv	9	*Protoambidensovirus*	*Densovirinae*	*Parvoviridae*
12	4mgu	9	*Scindoambidensovirus*	*Densovirinae*	*Parvoviridae*
11.8	3n7x	11	*Penstylhamaparvovirus*	*Hamaparvovirinae*	*Parvoviridae*
11.3	8er8	12	*Brevihamaparvovirus*	*Hamaparvovirinae*	*Parvoviridae*
5.6	7bgk	12	*Aparavirus*		*Dicistoviridae*
5.3	6shl	11	*Bacillarnavirus*		*Marnaviridae*
5.3	1b35	7	*Cripavirus*		*Dicistoviridae*
5.2	5lwg	11	*Aparavirus*		*Dicistoviridae*

**Table 4:** Comparative analysis of the Syngnathus scovelli chapparvoviorus’s multimer interfaces.

Interface	Buried Interface Area (Å^2^)	ΔiG (kcal/mol)
Dimer	751.9	−8
Trimer	2521.8	−27.1
Pentamer	1508.4	−19.3

## Data Availability

The obtained electron density map has been deposited to the EMDataResource under the accession number EMD-49314. The refined atomic model is available at the RCSB Protein Data Bank under the identification number of 9NEK.
